# Practical Enantioselective Reduction of Ketones Using Oxazaborolidine Catalysts Generated In Situ from Chiral Lactam Alcohols

**DOI:** 10.3390/molecules23102408

**Published:** 2018-09-20

**Authors:** Yasuhiro Kawanami, Ryo C. Yanagita

**Affiliations:** Department of Applied Biological Science, Faculty of Agriculture, Kagawa University, Miki-cho, Kagawa 761-0795, Japan; charlesy@ag.kagawa-u.ac.jp

**Keywords:** borane, reduction, asymmetric synthesis, enantioselective, lactam alcohol

## Abstract

Oxazaborolidine catalyst (CBS catalyst) has been extensively used for catalytic borane reduction with a predictable absolute stereochemistry and high enantioselectivity. However, the use of isolated CBS catalyst sometimes has the drawback of low reproducibility due to the aging of the CBS catalyst during storage. Therefore, we investigated a more reliable and practical method for the reduction of a variety of ketones including challenging substrates, primary aliphatic ketones, α,β-enones, and trifluoromethyl ketones. This review surveys the developments in borane reduction using oxazaborolidine catalysts generated in situ from chiral lactam alcohols and borane.

## 1. Introduction

**Figure 1:**
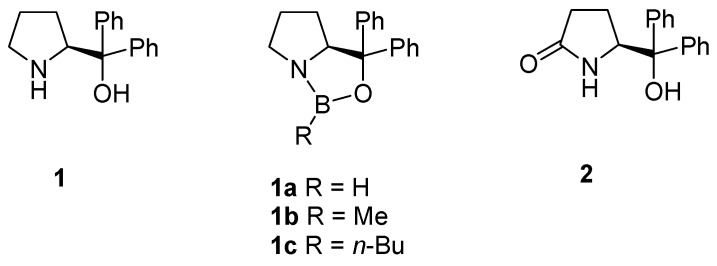
Various chiral catalysts and precatalysts.

**Scheme 1:**
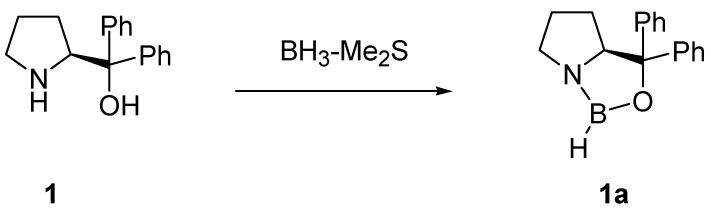
Generation of oxazaborolidine **1a** in situ from **1** and borane.

Asymmetric reduction of prochiral ketones is one of the most important methods for the synthesis of chiral secondary alcohols and constitutes a valuable step in the synthesis of a variety of natural products and several medicinally important compounds. The oxazaborolidine-catalyzed asymmetric borane reduction of prochiral ketones (CBS reduction) using chiral amino alcohols [1,2,3,4] has been extensively investigated, since the stoichiometric reductions were reported by Itsuno et al. [5,6,7] and the catalytic versions were reported by Corey et al. [8]. The chiral amino alcohol, (*S*)-α,α-diphenyl-2-pyrrolidinemethnol (**1**) derived from (*S*)-proline has been widely accepted as superior one that permits efficient synthesis of chiral secondary alcohols with predictable absolute stereochemistries (Figure 1). However, it has been described that the preparation of oxazaborolidine **1a** requires heating at reflux with excess BH_3_ in tetrahydrofuran (THF) [4]. On the other hand, Quallich et al. reported that the reaction of **1** with excess borane-dimethyl sulfide complex (BH_3_-Me_2_S) formed the oxazaborolidine **1a** in THF at room temperature for 8–10 h (Scheme 1) [9]. Later, Yanagi et al. also reported that the catalyst **1a** was generated from **1** and BH_3_-Me_2_S in THF, ether, and hexane at room temperature for 1 h [10]. Although the B-Me oxazaborolidine **1b** formed by the reaction of **1** with methylboronic acid has been developed as an air- and moisture- stable catalyst that catalyzes the borane reduction of ketones with an excellent enantioselectivity [11], there is the requirement of complete removal of water to avoid undesired effects [12]. As part of our studies in asymmetric synthesis, we have investigated a more convenient and practical method for the reduction of a variety of ketones. This review surveys the developments in borane reduction using in situ generated oxazaborolidine catalysts from chiral lactam alcohols and borane over the last fifteen years. Furthermore, modifications of the method are described for the borane reduction of challenging substrates, i.e., primary aliphatic ketones, α,β-enones and trifluoromethyl ketones.

## 2. Oxazaborolidine Catalyst Generated In Situ from a Chiral Lactam Alcohol and Borane

**Scheme 2:**
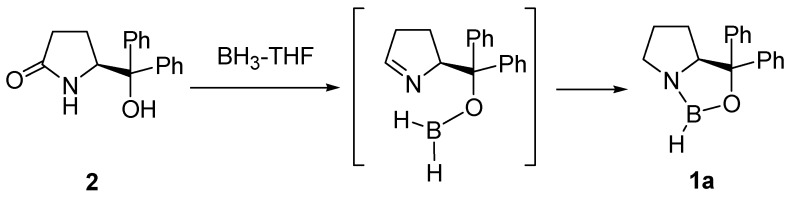
Generation of oxazaborolidine **1a** in situ from **2** and borane.

To overcome certain drawbacks, such as difficulties in handling and the reproducibility of the isolated oxazaborolidine catalysts, an alternative method was needed. We considered that the chiral lactam alcohol **2** [13], (*S*)-5-(diphenylhydroxymethyl)pyrrolidin-2-one, could be rapidly reduced with borane to the corresponding imine, which would be further reduced by the neighboring alkoxyborane to form the oxazaborolidine **1a** (Scheme 2). This was inferred from the report that the racemic amino alcohol **1** could be prepared from the racemic lactam alcohol **2** through its reduction with borane [10].

**Figure 2:**
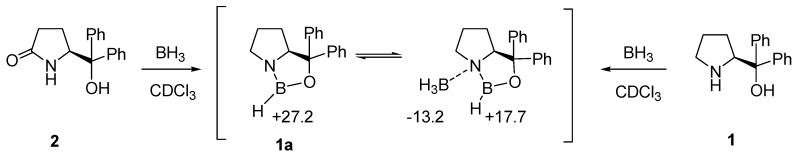
^11^B NMR shifts.

Therefore, we conducted ^11^B NMR analysis of the in situ generated oxazaborolidine catalyst as shown in Figure 2. The ^11^B NMR (CDCl_3_) spectrum indicated the presence of the oxazaborolidine complex with borane (+17.7 ppm and −13.2 ppm) and free oxazaborolidine (+27.2 ppm) [14]. These chemical shifts were mostly consistent with those of the oxazaborolidine **1a** generated in situ from (*S*)-diphenylpyrrolidinemethanol and borane [10] under the same conditions.

As a result, we expected that the oxazaborolidine **1a** generated in situ from **2** and borane should possibly catalyze the reduction of prochiral ketones with high enantioselectivities.

## 3. Asymmetric Reduction of Ketones

**Scheme 3:**
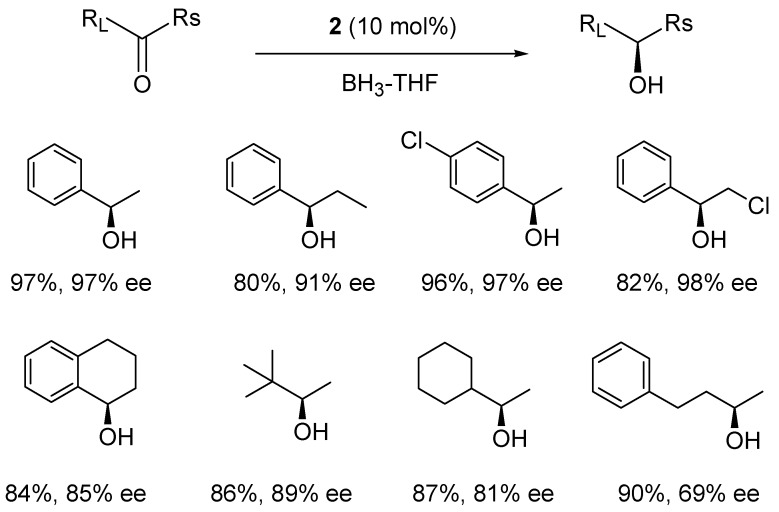
Asymmetric reduction of ketones using chiral lactam alcohol **2**. All reactions were carried out with 10 mol% of **2** and 1.0 equiv of BH_3_ in THF at room temperature.

The lactam alcohol **2** was readily prepared by the reaction of phenylmagnesium bromide with methyl (*S*)-pyroglutamate as reported previously [13]. The reduction of the chiral lactam alcohol **2** (10 mol%) with 1 equivalent of BH_3_-THF smoothly proceeded at room temperature within 5 min. We found that the resulting oxazaborolidine intermediate catalyzed the borane reduction of various ketones, affording chiral secondary alcohols in good yields and enantiomeric excess (ee). The results are summarized in Scheme 3 [15].

The reduction of aryl methyl, ethyl, and chloromethyl ketone with borane and 10 mol% of **2** afforded the corresponding (*R*)-secondary alcohols, except for (*S*)-2-chloro-1-phenylethanol, with excellent enantioselectivities (91–98% ee). α-Tetralone, a cyclic aryl ketone, was reduced with good enantioselectivity (85% ee). The reduction of alkyl methyl ketones having a tertiary, secondary, and primary alkyl group proceeded with good to moderate enantioselectivities (89% ee, 81% ee, and 69% ee, respectively). Thus, the yields and enantioselectivities of the reduction using the lactam alcohol **2** and borane were comparable to those of the isolated catalyst **1a**. Moreover, **1** could be obtained in good yield by extraction from aqueous acidic solution after quenching, suggesting that the lactam alcohol **2** was reduced by borane to actually generate the catalyst **1a** in situ at room temperature in a short time.

## 4. Asymmetric Reduction of Aliphatic Ketones

Although the reduction of most aromatic ketones proceeded with excellent enantioselectivities, those of aliphatic ketones, in particular primary aliphatic ketones, usually afforded moderate enantioselectivities. For example, the enantioselectivity for the reduction of benzylacetone using **1a** generated in situ from the chiral lactam alcohol **2** and borane was only 69% ee (that of Me-CBS **1b** was 64% ee under the same reaction condition). Accordingly, we decided to modify the catalytic borane reduction using chiral lactam alcohol **2** to improve the enantioselectivity of the reduction of aliphatic ketones which are challenging substrates [16].

**Figure 3:**
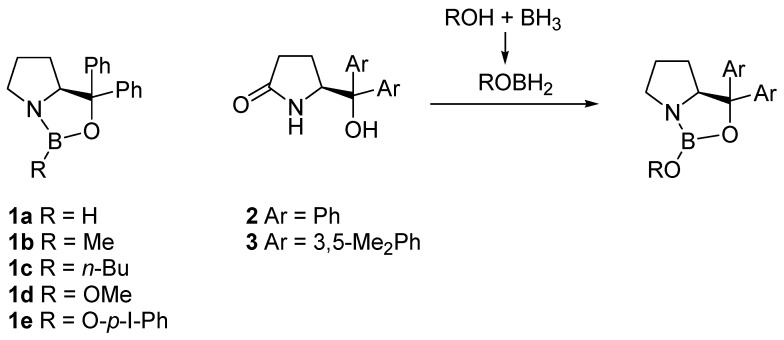
Various chiral catalysts and precatalysts.

**Scheme 4:**
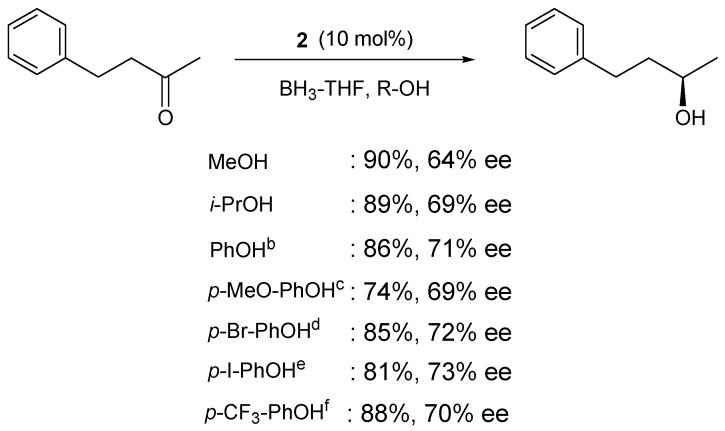
Asymmetric reduction ^a^ of benzylacetone using chiral lactam alcohol **2**. ^a^ All reactions were carried out with 10 mol% of **2** and 1.2 equiv of BH_3_ and ROH in THF at room temperature. ^b^ p*K*_a_ 9.99. ^c^ p*K*_a_ 10.10. ^d^ p*K*_a_ 9.17. ^e^ p*K*_a_ 9.21. ^f^ p*K*_a_ 8.68.

Shioiri et al. reported that trimethyl borate improved the reactivity and enantioselectivity of CBS reduction through the B-OMe oxazaborolidine **1d** [17]. Therefore, we expected that the electronic effects of the boron substituent of the oxazaborolidine should enhance the reactivity and enantioselectivity. First, BH_3_-THF solution was added dropwise to various alcohols in THF and stirred for 20 min at room temperature. During this period, the generation of H_2_ was observed. Then, the chiral lactam alcohol **2** (10 mol%) was added to the alkoxyborane solution and stirred further for 1 h (Figure 3). Slow addition of benzylacetone as an aliphatic ketone to the catalyst generated in situ for 1 h afforded a (*R*)-secondary alcohol in good yields (74–90%, Scheme 4). While addition of neither methanol nor 2-propanol improved ee (69% ee, without the alcohols), the addition of substituted phenols afforded equal to slightly higher enantioselectivities (69–73% ee). We found a nonlinear dependence of enantioselectivity on the p*K*_a_ of these phenols [18]. The most acidic *p*-(trifluoromethyl)phenol did not provide superior results (70% ee) and *p*-iodophenol provided the highest result (73% ee). These results suggest that *p*-iodophenoxy oxazaborolidine **1e** has appropriate properties to increase the reactivity and enantioselectivity, though the reaction mechanism is very complicated due to possible involvement of several intermediate borane species. When *p*-iodophenol was added to the in situ generated **1a**, the enantioselectivity decreased from 73% ee to 67% ee. This result indicated that the reduction of **2** with *p*-iodophenoxyborane should generate B-OAr oxazaborolidine catalyst **1e**, though the detection of **1e** by ^11^B NMR analysis has not been successful, possibly due to its instability.

**Scheme 5:**
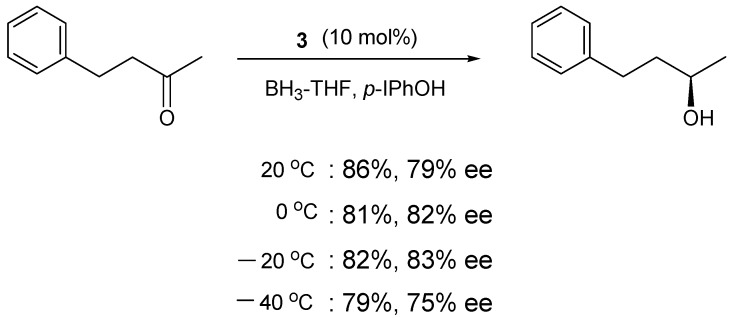
Effect of temperature on enantioselectivity for the reduction of benzylacetone using **3** and *p*-iodophenoxyborane. All reactions were carried out with 10 mol% of **3** and 1.2 equiv of BH_3_ and *p*-iodophenol in THF at room temperature.

The screening of new chiral lactam alcohols with the 4-substituted or 3,5-disubstituted phenyl of the carbinol center revealed that the use of **3** with 3,5-dimethylphenyl improved the enantioselectivity from 73 to 79% ee. A nonlinear temperature effect on the enantioselectivity was also observed (Scheme 5). When the reaction temperature decreased from 20 °C to −20 °C, the enantioselectivity gradually increased up to 83% ee, and then dropped to 75% ee at −40 °C. This temperature effect contrasts with the reported result that general CBS reduction at lower temperature yielded lower enantioselectivities [19]. Therefore, we confirmed that the reduction with **3** (10 mol%) and BH_3_ (1.2 equiv) at −20 °C resulted in a decrease in enantioselectivity, 35% ee, implying that a new oxazaborolidine catalyst **1e** would generate from **3** and *p*-iodophenoxyborane.

**Scheme 6:**
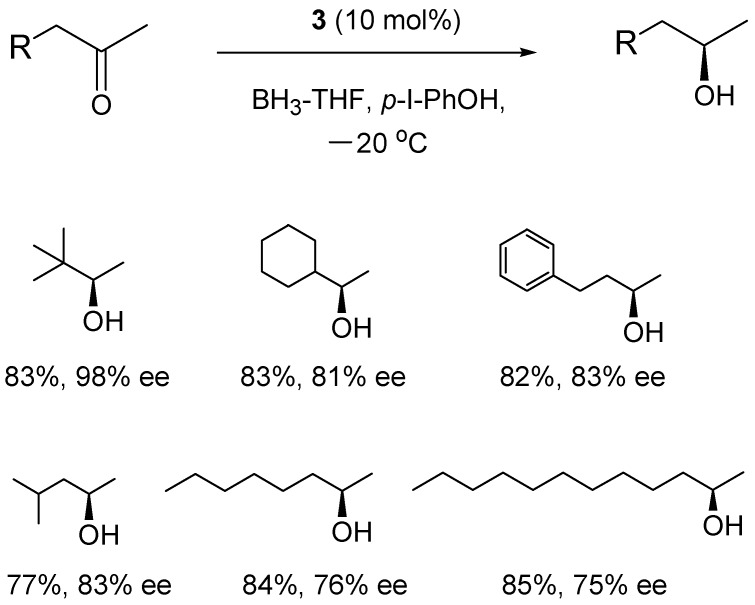
Asymmetric reduction of aliphatic ketones using the chiral lactam alcohol **3** and *p*-iodophenoxyborane. All reactions were carried out with 10 mol% of **3** and 1.2 equiv of BH_3_ and *p*-iodophenol in THF at −20 °C.

Under these optimized reaction conditions, the reduction of alkyl methyl ketones having a tertiary, secondary, and primary alkyl group with **3** and *p*-iodophenoxyborane proceeded with excellent to good enantioselectivities (98% ee, 81% ee, and 83% ee, respectively; Scheme 6). The reduction of cyclohexyl methyl ketone at room temperature proceeded with a better enantioselectivity (90% ee) contrary to that involving the other ketones, suggesting that the temperature effect is somewhat substrate-specific.

## 5. Asymmetric Reduction of α,β-Enones

**Scheme 7:**
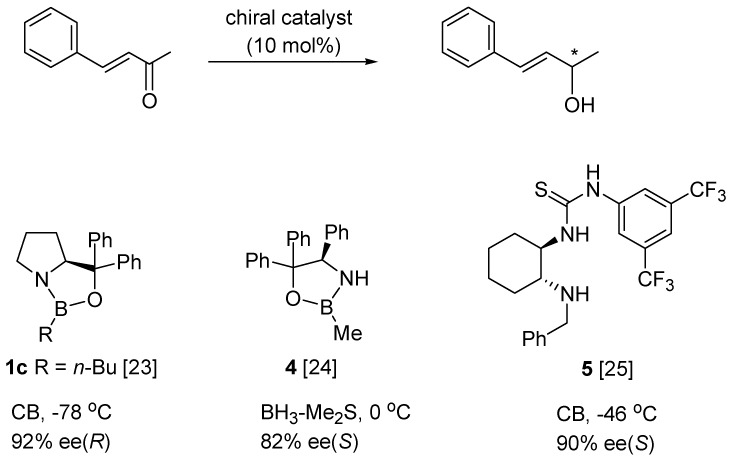
Chiral catalysts for the borane reduction of benzalacetone.

Chiral allylic alcohols are important natural products [11] and the key intermediates for many stereospecific reactions, such as Claisen rearrangement [20], epoxidation [21], and S_N_2′ displacement with organometallic regents [22]. However, the reports concerning oxazaborolidine catalysis for the reduction of α,β-enones are very limited, probably due to the occurrence of a side reaction, i.e., hydroboration. For example, Corey et al. reported that the reduction of benzalacetone with Bu-CBS **1c** (10–15 mol%) and 2 equiv. of catecholborane (CB) in toluene at −78 °C proceeded with excellent enantioselectivity (92% ee) [23], as shown in Scheme 7. On the other hand, Bach et al. described that the reduction of benzalacetone using **4** and BH_3_-Me_2_S in THF at 0 °C provided a lower enantioselectivity (82% ee) [24]. Recently, Falck et al. also reported the enantioselective reduction of benzalacetone using CB and air-stable bifunctional thiourea-amine organocatalyst **5**, instead of CBS oxazaborolidine catalyst, with a high enantioselectivity (90% ee) [25]. In this context, we investigated the enantioselective reduction of α,β-enones as challenging substrates using the oxazaborolidine generated in situ from the chiral lactam alcohol **3** to expand its scope and generality [26].

We first examined the effect of the alcohol on the enantioselectivity of the reduction of benzalacetone as a substrate using the chiral lactam alcohol **3** (10 mol%) and BH_3_-THF at 0 °C because the reduction with **3** and BH_3_-THF afforded a complicated mixture as a result of the hydroboration side reaction. We found that the addition of *p*-iodophenol significantly increased the enantioselectivity compared to 2-propanol [27,28] and *p*-halogen substituted phenols as shown in Table 1. Then, we examined the solvent effect in the reduction with **3** and *p*-iodophenoxyborane. Toluene afforded a higher yield than THF and the polar solvents, dichloromethane (CH_2_Cl_2_) and chloroform (CHCl_3_), afforded somewhat lower enantioselectivities (56% ee and 66% ee).

Stone demonstrated that Ph-CBS catalyst is more sensitive to temperature change than Me- and Bu-CBS catalysts and indicated that CBS reduction may be optimized to obtain the highest selectivity possible for a given catalyst and ketone by adjusting the temperature [19]. Therefore, we examined the temperature effect on enantioselectivity for the reduction of benzalacetone using the in situ generated *p*-I-PhO-oxazaborolidine catalyst **1e**.

**Scheme 8:**
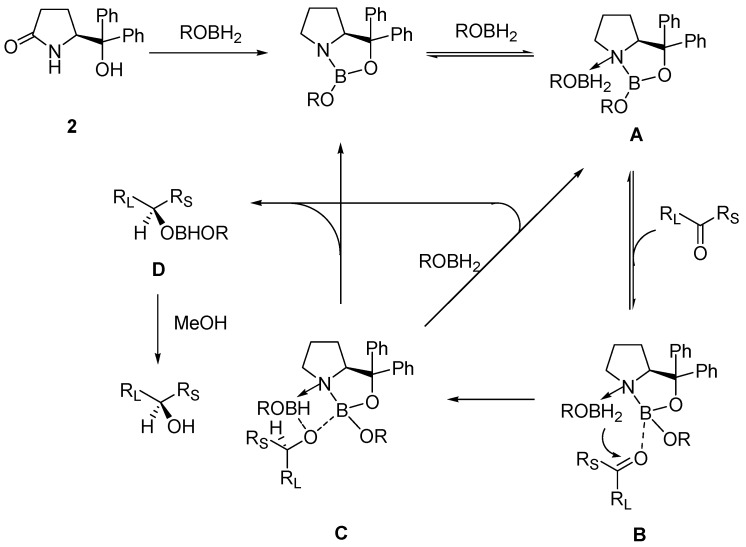
Mechanism of the CBS reduction of ketones, proposed by Core et al.

When the reaction temperature is lowered from 0 °C to −60 °C, the enantioselectivity increased up to 84% ee at −40 °C, and then dropped to 63% ee at −60 °C (Table 2). Therefore, the optimal temperature in toluene was found to be −40 °C. The temperature effect might be attributed to the acceleration of the catalytic cycle as a result of the dissociation of the product **D** and the regeneration of the catalyst from the reaction intermediate **C** (Scheme 8). Interestingly, we observed that the enantioselectivity was susceptible to the balance between the borane equivalents and catalyst loading. When 8 mol% of the chiral lactam alcohol **3** and 1.0 equiv. of *p*-I-PhOBH_2_ were used, the enantioselectivity increased up to 90% ee.

**Figure 4:**
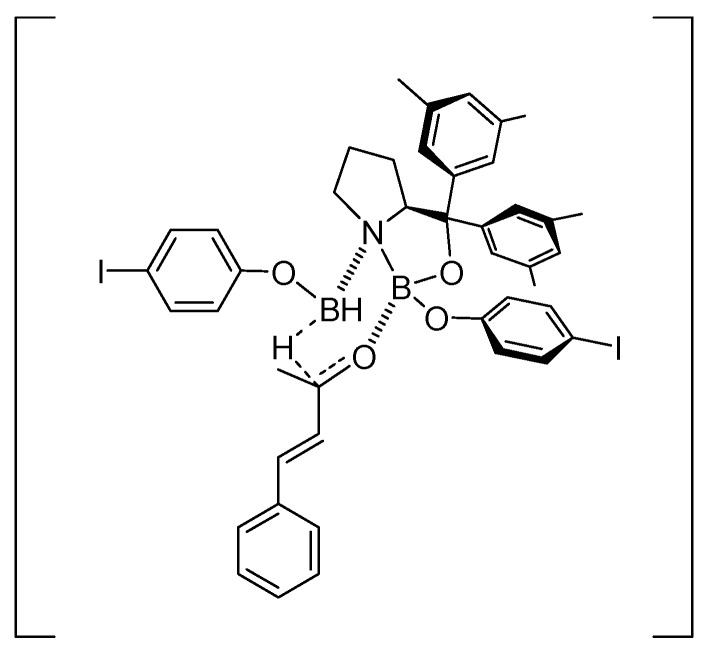
Proposed transition state model.

The absolute configuration of the resulting secondary alcohol was (*R*), which agreed with the reduction process catalyzed by Bu-CBS **1c** [23]**.** The enantioselectivity was proposed to originate via a six-membered transition state **B** shown in Scheme 8 [4]. Therefore, this result can be explained by a transition state model (Figure 4) in which the olefinic part behaves as the large group R_L_ and the hydride of 4-I-PhOBH_2_ would attack on the *Si*-face of the carbonyl carbon of benzalacetone to give the (*R*)-alcohol.

**Scheme 9:**
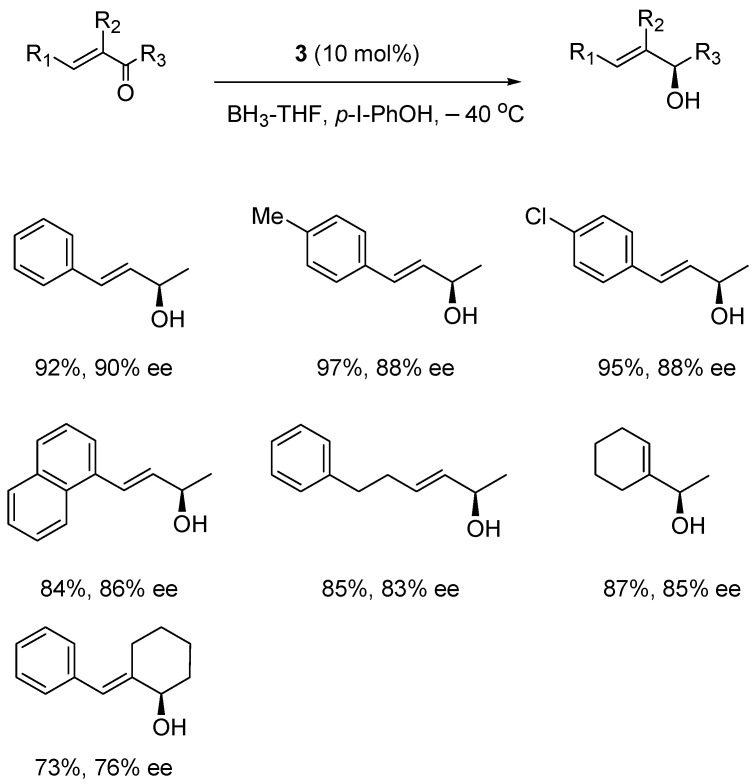
Asymmetric reduction of α,β-enones using **3** and *p*-iodophenoxyborane. All reactions were carried out with 10 mol% of **3** and 1.2 equiv of BH_3_ and *p*-iodophenol in THF at −40 °C.

Under the optimized conditions, we investigated the borane reduction of various α,β-enones to evaluate the scope and limitations of the substrate. As shown in Scheme 9, the reduction of *p*-chloro and *p*-methyl substituted benzalacetones proceeded with a slightly lower enantioselectivity than that of benzalacetone, regardless of their electronic properties. The bulkier 4-(1-naphtyl)-3-butene-2-one was reduced with 86% ee. The reduction of 6-phenyl-3-buten-2-one proceeded with a slightly lower enantioselectivity (83% ee) compared to that of benzalacetone, possibly due to the lack of conjugation with the benzene ring. Although the reduction of the cyclic enone, 1-acetylcyclohexene afforded a high enantioselectivity (85% ee), the reduction of the exocyclic enone, phenylmethylidenecyclohexanone, revealed a moderate enantioselectivity (76% ee). This result can be explained by the less stable *s-cis* conformation of the exocyclic enone, unlike the *s-trans* conformation of the cyclic enone and other enones of the transition state (Figure 4).

## 6. Asymmetric Reduction of Trifluoromethyl Ketones

**Scheme 10:**
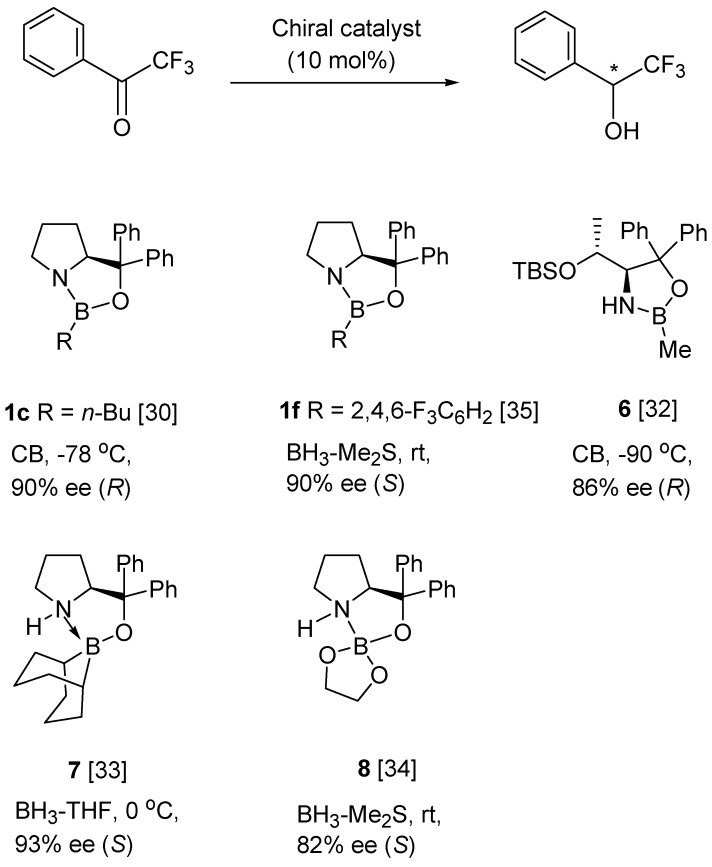
Various chiral catalysts for the borane reduction of 2,2,2-trifluoroacetophenone.

Fluorine-containing chiral alcohols have recently been studied as potentially good precursors for preparing ferroelectric liquid crystals [29]. However, because of their high reactivity, the oxazaborolidine-catalyzed asymmetric borane reduction of trifluoromethyl ketones usually affords the corresponding chiral secondary alcohols with a poor enantioselectivity [4], except for the reduction of the trifluoromethyl ketones with Bu-CBS **1c** and CB [30,31]. This low enantioselectivity can be explained by the low coordinating ability of the carbonyl oxygen to the oxazaborolidine catalyst and the noncatalytic reduction with borane. It was also reported that the oxazaborolidine **6** derived from l-threonine and CB at −90 °C afforded high enantioselectivities [32]. On the other hand, other methods using the catalyst prepared from (*S*)-diphenylpyrrolidinemethanol with 9-borabicyclo[3.3.1]nonane (9-BBN) **7** [33], spiroborate ester **8** [34], and electronically tuned-CBS catalyst **1f** with high Lewis acidity [35] have been reported with good to high enantioselectivities and, interestingly, stereochemistry opposite to that of **1c** (Scheme 10). Therefore, we set out to investigate the catalytic asymmetric reduction of trifluoromethyl ketones using the simple oxazaborolidine **1a** generated in situ from the chiral lactam alcohol **2** and borane [14,36] to clarify its scope and enantioselection.

We first examined the effect of borane reagents on enantioselectivity for the reduction of trifluoroacetophenone as shown in Table 3. Various borane reagents, BH_3_-THF, BH_3_-Me_2_S, CB, and *p*-I-PhOBH_2_ were used at room temperature. Although the reduction with BH_3_-Me_2_S or CB resulted in small enantioselectivities and the use of *p*-I-PhOBH_2__,_ which was effective for the reduction of aliphatic ketones [17], did not improve the enantioselectivity, the use of BH_3_-THF was found to be optimal as a reducing borane reagent for our method. Further study of the solvent revealed that the polar solvent CHCl_3_ afforded a significantly higher enantioselectivity (up to 80% ee) for the reduction with **2** and BH_3_-THF. However, we observed variable enantioselectivities depending on the storage period of BH_3_-THF. To our surprise, when a new commercially available BH_3_-THF (stabilized with ca. 0.005 M NaBH_4_) was employed, only lower enantioselectivities (55% ee) were obtained. These results imply that a properly degraded BH_3_-THF is superior to the one containing NaBH_4_-stabilizer possibly due to the butoxyborane species produced by the reduction of THF with BH_3_-THF during storage, as reported by Nettles et al. [37]. They also described that the addition of a Lewis acid deactivated the NaBH_4_ stabilizer in BH_3_-THF and that the BF_3_-THF complex (3–8 mol%) proved to be the best with a high enantioselectivity for the reduction of acetophenone among the examined Lewis acids. Fu et al. simultaneously reported that the chemo- and enantioselectivities dramatically increased when using an acid (5 mol% of BF_3_-OEt_2_ or *p*-toluenesulfonic acid) as a scavenger of the NaBH_4_ stabilizer in BH_3_-THF for the reduction of a ketone having the chiral 4-phenyl-2-oxazolidinone auxiliary [38]. Therefore, we expected that addition of BF_3_ could enhance the enantioselectivity for the reduction of trifluoroacetophenone with the oxazaborolidine **1a**. The reduction with **2** (10 mol%) and BH_3_-THF (0.8 equiv.) in the presence of BF_3_ (8 mol%) provided the (*S*)-alcohol in 85% yield, with a higher enantioselectivity (60% ee) compared to that without BF_3_, implying that the BF_3_ remaining after scavenging the NaBH_4_ stabilizer might enhance the enantioselectivity. Accordingly, we carefully examined the effect of BF_3_ loading on the enantioselectivity, which was not described in the papers mentioned above ([37,38]). The enantioselectivity increased depending on the BF_3_ loading and reached 80% ee at 160 mol%, which was found to be the best loading after screening for the optimal loading. To clarify the effect of its addition, 8 and 160 mol% of BF_3_ were added to the reduction process using the pure BH_3_ generated in situ from tetra-*n*-butylammonium borohydride (TBAB) and methyl iodide (MeI) [39], thus producing the (*S*)-alcohol in high yield and with high enantioselectivities. These results suggested that excess BF_3_-THF not only deactivated NaBH_4_ stabilizer, but also actually improved the enantioselectivity.

**Figure 5:**
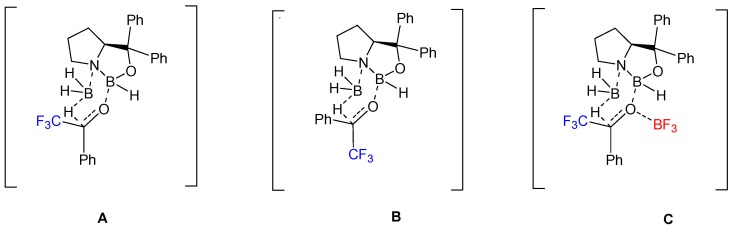
Proposed transition state models.

The stereochemistry of the resulting secondary alcohol during the reduction with BH_3_-THF was the same (*S*)-configuration as that observed for the reductions catalyzed by **1f** [35], **7** [33], and **8** [34], suggesting that the hydride attack on the *Re*-face of the carbonyl group of trifluoroacetophenone might occur via a typical transition state (Figure 5A) for general CBS reductions.

Corey et al. proposed another transition state (Figure 5B) for the reverse (*R*)-enantioselection occurring hydride attack on the *Si*-face of the carbonyl group during the reduction with Bu-CBS **1c** and CB at −78 °C due to the electrostatic repulsion between the trifluoromethyl group and the lone pair of the carbonyl group. To clarify whether the addition of BF_3_ causes a change in the oxazaborolidine catalyst, we carried out ^11^B NMR study of the in situ generated oxazaborolidine catalyst in the presence of BF_3_. The ^11^B NMR analysis revealed that the major signals (+17.7 ppm and −13.2 ppm) of the oxazaborolidine complex with BH_3_-THF and free oxazaborolidine (−27.2 ppm) did not change upon the addition of BF_3_-THF to the catalyst solution. Thus, an interaction between the oxazaborolidine catalyst and BF_3_-THF was deemed to be negligible, if any. These results suggested that BF_3_ might not coordinate to the in situ generated catalyst, but rather coordinate to trifluoroacetophenone, in an *anti*-relationship to the trifluoromethyl group, thereby stabilizing the complex in the transition state (Figure 5C).

**Scheme 11:**
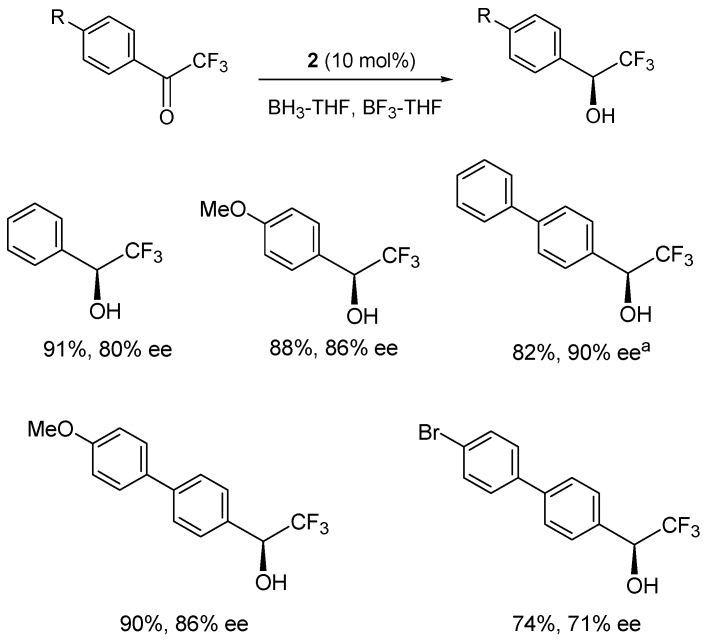
Asymmetric reduction of various trifluoromethyl ketones using **2**. All reactions were carried out with 10 mol% of **2**, 160 mol% of BF_3_ and 0.8 equiv of BH_3_ in CHCl_3_ at room temperature. ^a^ Temperature, 40 °C.

Under the optimized reaction conditions, the reduction of aromatic trifluoromethyl ketones including the para-substituted biphenyl trifluoromethyl ketones proceeded with moderate to high enantioselectivities as shown in Scheme 11. The electron-donating methoxy and phenyl groups of the benzene ring increased the enantioselectivity to 86 and 90% ee, respectively. The reduction of the biphenyl trifluoromethyl ketone with the methoxy group afforded the (*S*)-alcohol in 90% yield with the enantioselectivity of 86% ee, which is similar to that obtained from the reduction with Bu-CBS **1c** or the oxazaborolidine derived from l-threonine and CB at −90 °C, but the opposite (*S*)-configuration [33]. On the other hand, the biphenyl trifluoromethyl ketone with an electron-withdrawing bromo group was reduced with modest enantioselectivity (71% ee).

## 7. Conclusions

We have demonstrated that the oxazaborolidine catalysts generated in situ from the chiral lactam alcohol **2** and borane catalyzed the enantioselective reduction of aromatic ketones with high enantioselectivities. Furthermore, in the case of aliphatic ketones and α,β-enones, the use of *p*-iodophenoxyborane improved the enantioselectivities at low temperatures and, in the case of trifluoromethyl ketones, the addition of BF_3_ enhanced the enantioselectivities at room temperature. These modified methods offer good reproducibility and render the catalytic borane reductions more practical because these catalysts can be easily generated in situ from stable chiral lactam alcohols and borane before use.

## Figures and Tables

**Table 1:**
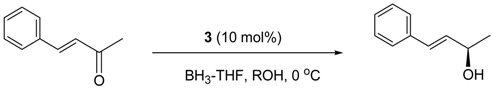
Asymmetric reduction of α,β-enones using the chiral lactam alcohol **3** ^a^.

ROH	Solvent	Yield	ee (%)
*i*-Pr	THF	3	7
Ph	THF	7	8
*p*-Cl-Ph	THF	20	46
*p*-Br-Ph	THF	30	55
*p*-I-Ph	THF	48	73
*p*-I-Ph	toloene	61	73
*p*-I-Ph	CH_2_Cl_2_	13	56
*p*-I-Ph	CH_2_Cl_2_	43	66

^a^ All reactions were carried out with 10 mol% of **3** and 1.2 equiv. of the alcohol and BH_3_-THF at 0 °C for 2 h.

**Table 2:**
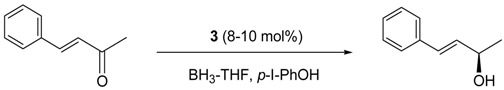
Effect of temperature, ligand loading, and borane equivalent on enantioselectivity for the reduction of benzalacetone using chiral lactam alcohol **3** and *p*-iodophenoxyborane ^a^.

Temp. (°C)	3 (mol%)	*p*-I-PhOBH_2_ (equiv)	Yield (%)	ee (%)
0	10	1.2	61	73
−20	10	1.2	66	81
−40	10	1.2	87	84
−60	10	1.2	66	63
−40	8	1.0	92	90

^a^ All reactions were carried out with 10 mol% of **3** and 1.2 equiv of BH_3_ and *p*-iodophenol in THF.

**Table 3:**
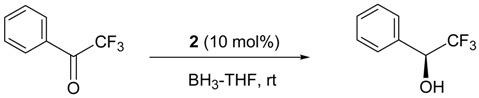
Asymmetric reduction of 2,2,2-trifluoroacetophenone using lactam alcohol **2** ^a^.

Reducing Agent	Solvent	BF_3_ (mol%)	Yield (%)	ee (%)
BH_3_-THF	THF	–	89	52
BH_3_-Me_2_S	THF	–	77	3
CB	THF	–	36	2
*p*-I-PhOBH_2_	THF	–	67	42
BH_3_-THF	toluene	–	82	56
BH_3_-THF	CH_2_Cl_2_	–	97	78
BH_3_-THF	CHCl_3_	–	90	80
BH_3_-THF ^b^	CHCl_3_	–	94	55
BH_3_-THF ^b^	CHCl_3_	8	85	60
BH_3_-THF ^b^	CHCl_3_	160	91	80
TBAB/MeI ^c^	CHCl_3_	8	94	67
TBAB/MeI ^c^	CHCl_3_	160	97	81

^a^ All reactions were carried out with 10 mol% of **2** and 1.2 equiv of BH_3_ and *p*-iodophenol in THF at −20 °C. ^b^ New bottle containing approximately 0.005M NaBH_4_. ^c^ Pure BH_3_ was generated in situ.

## References

[1] Wallbaum S., Martens J. (1992). Asymmetric syntheses with chiral oxazaborolidines. Tetrahedron Asymmetry.

[2] Singh V.K. (1992). Practical and useful methods for the enantioselective reduction of unsymmetrical ketones. Synthesis.

[3] Deloux L., Srebnik M. (1993). Asymmetric boron-catalyzed reactions. Chem. Rev..

[4] Corey E.J., Helal C.J. (1998). Reduction of carbonyl compounds with chiral oxazaborolidine catalysts: A new paradigm for enantioselective catalysis and a powerful new synthetic method. Angew. Chem. Int. Ed..

[5] Hirao A., Itsuno S., Nakahama S., Yamazaki N. (1981). Asymmetric reduction of aromatic ketones with chiral alkoxy-amine-borane complexes. J. Chem. Soc. Chem. Commun..

[6] Itsuno S., Hirao A., Nakahama S., Yamazaki N. (1983). Asymmetric synthesis using chirally modified borohydrides. Part 1. Enantioselective reduction of aromatic ketones with the reagent prepared from borane and (*S*)-valinol. J. Chem. Soc. Perkin Trans. 1.

[7] Itsuno S., Ito A., Hirao A., Nakahama S. (1984). Asymmetric reduction of aliphatic ketones with reagent prepared from (*S*)-(−)-2-amino-3-methyl-1,1-diphenylbutan-1-ol and borane. J. Org. Chem..

[8] Corey E.J., Bakshi R.K., Shibata S. (1987). Highly enantioselective borane reduction of ketones catalyzed by chiral oxazaborolidines. Mechanism and synthetic implications. J. Am. Chem. Soc..

[9] Qualich G., Woodall T.M. (1993). In situ oxazaborolidines, practical enantioselective hydride reagents. Synlett.

[10] Yanagi T., Kikuchi K., Takeuchi H., Ishikawa T., Nishihara T., Kubota M., Yamamoto I. (2003). Asymmetric borane reduction of prochiral ketone using chiral bis (α,α-diphenyl-2-pyrrolidinemethanol) carbonate. Chem. Pharm. Bull..

[11] Corey E.J., Bakshi R.K., Shibata S., Chen C.-P., Singh V.K. (1987). A stable and easily prepared catalyst for the enantioselective reduction of ketones. Application to multistep syntheses. J. Am. Chem. Soc..

[12] Mathre D.J., Jones T.K., Xavier L.C., Blacklock T.J., Reamer R.A., Mohan J.J., Jones E.T.T., Hoogsteen K., Baum M.W., Grabowski E.J.J. (1991). A practical enantioselective synthesis of α,α-diaryl-2-pyrrolidinemethanol. Preparation and chemistry of the corresponding oxazaborolidines. J. Org. Chem..

[13] Fujihara H., Tomioka K. (1999). Asymmetric protonation of lithium enolate using 5-substituted pyrrolidin-2-one as a chiral proton source. J. Chem. Soc. Perkin Trans. 1.

[14] Harauchi Y., Takakura C., Furumoto T., Yanagita R.C., Kawanami Y. (2015). Effect of BF_3_ on the enantioselective reduction of trifluoromethyl ketones using a chiral lactam alcohol with borane. Tetrahedron Asymmetry.

[15] Kawanami Y., Murao S., Ohga T., Kobayashi N. (2003). Practical enantioselective reduction ketones using oxazaborolidine catalyst generated in situ from chiral lactam alcohol and borane. Tetrahedron.

[16] Kawanami Y., Mikami Y., Hoshino K., Suzue M., Kajihara I. (2009). Enantioselective reduction of aliphatic ketones using oxazaborolidine catalyst generated in situ from chiral lactam alcohol and phenoxyborane. Chem. Lett..

[17] Masui M., Shioiri T. (1997). A practical method for asymmetric borane reduction of prochiral ketones using amino alcohols and trimethylborate. Synlett.

[18] Tehan B.G., Lloyd E.J., Wong M.G., Pitt W.R., Montana J.G., Manallack D.T., Gancia E. (2002). Estimation of pKa using semiempirical molecular orbital methods. Part 1: Application to phenols and carboxylic acids. Mol. Inf..

[19] Stone G. (1994). Oxazaborolidine catalyzed borane reductions of ketones: A significant effect of temperature on selectivity. Tetrahedron Asymmetry.

[20] Gunes Y., Polat M.F., Sahin E., Fleming F.F., Altundas R. (2010). Enantioselective synthesis of cyclic, quaternary oxonitriles. J. Org. Chem..

[21] Corey E.J., Su W.-G. (1988). Enantioselective total synthesis of bilobalide, A C15 ginkgolide. Tetrahedron Lett..

[22] Calaza M.I., Hupe E., Knochel P. (2003). Highly *anti*-selective S_N_2′ substitutions of chiral cyclic 2-iodo-allylic alcohol derivatives with mixed zinc-copper reagents. Org. Lett..

[23] Corey E.J., Bakshi R.K. (1990). A new system for catalytic enantioselective reduction of achiral ketones to chiral alcohols. Synthesis of chiral α-hydroxy acids. Tetrahedron Lett..

[24] Bach J., Berenguer R., Farràs J., Garcia J., Meseguer J., Vilarrasa J. (1995). Allylic alcohols of unexpected configuration by oxazaborolidine-catalyzed reduction of α,β-unsaturated ketones. An explanation based on MO calculations. Tetrahedron Asymmetry.

[25] Li D.R., He A., Falck J.R. (2010). Enantioselective, organocatalytic reduction of ketones using bifunctional thiourea-amine catalysts. Org. Lett..

[26] Kawanami Y., Mikami Y., Kiguchi K., Harauchi Y., Yanagita R.C. (2011). Enantioselective reduction α,β-enones using an oxazaborolidine catalyst generated in situ from chiral lactam alcohol. Tetrahedron Asymmetry.

[27] Shi Y., Cai D., Dolling U.-H., Douglas A.W., Tschaen D.M., Verhoeven T.R. (1994). An improved method for chiral oxazaborolidine-catalyzed reduction of 4-chromanone analogs and MK-0499. Tetrahedron Lett..

[28] Tschaen D.M., Abramson L., Cai D., Desmond R., Dolling U.-H., Frey L., Karady S., Shi Y., Verhoeven T.R. (1995). Asymmetric synthesis of MK-0499. J. Org. Chem..

[29] Dolbier W.R. (2005). Fluorine chemistry at the millennium. J. Fluorine Chem..

[30] Corey E.J., Cheng X., Cimprich K.A., Sarshar S. (1991). Remarkably effective and simple syntheses of enantiomerically pure secondary carbinol from achiral ketones. Tetrahedron Lett..

[31] Corey E.J., Link J.O., Bakshi R.K. (1992). A mechanistic and structural analysis of the basis for high enantioselectivity in the oxazaborolidine-catalyzed reduction of trihalomethyl ketones by catecholborane. Tetrahedron Lett..

[32] Fujisawa T., Onogawa Y., Shimizu M. (1998). Structural effect of the oxazaborolidine derived from L-threonine in the reduction of (trifluoroacetyl)biphenyl derivatives with catecholborane. Tetrahedron Lett..

[33] Kanth J.V.B., Brown H.C. (2002). A new catalytic enantioselective reducing reagent system from (−)-α,α-diphenylpyrrolidinemethanol and 9-borabicyclo[3.3.1]nonane, especially effective for hindered and substituted aralkylketones. Tetrahedron.

[34] Stepanenko V., Jesus M.D., Correa W., Guzman I., Vazquez C., Cruz W., Ortiz-Marciales M., Barnes C.L. (2007). Enantioselective reduction of prochiral ketones using spiroborate esters as catalysts. Tetrahedron Lett..

[35] Korenaga T., Nomura K., Onoue K., Sakai T. (2010). Rational electronic tuning of CBS catalyst for highly enantioselective borane reduction of trifluoroacetophenone. J. Chem. Soc. Chem. Commun..

[36] Kawanami Y., Hoshino K., Tsunoi W. (2011). Enantioselective reduction of trifluoromethyl ketones using an oxazaborolidine catalyst generated in situ from chiral lactam alcohol. Tetrahedron Asymmetry.

[37] Nettles A.M., Matos K., Burkhardt E.R., Rouda D.R., Corella J.A. (2002). Role of NaBH_4_ stabilizer in the oxazaborolidine-catalyzed asymmetric reduction of ketones with BH_3_-THF. J. Org. Chem..

[38] Fu X., McAllister T.L., Thiruvengadam T.K., Tann C.-H., Su D. (2003). Process for preparing ezetimibe intermediate by an acid enhanced chemo- and enantioselective CBS catalyzed ketone reduction. Tetrahedron Lett..

[39] Anwar S., Periasamy M. (2006). A convenient method for the preparation of oxazaborolidine catalyst in situ using (*S*)-α,α-diphenylpyrrolidinemethanol, tetrabutylammonium borohydride, and methyl iodide for the asymmetric reduction of prochiral ketones. Tetrahedron Asymmetry.

